# Meaningful consumer involvement in cancer care: a systematic review on co-design methods and processes

**DOI:** 10.1093/jncics/pkae048

**Published:** 2024-06-19

**Authors:** Nicole Kiss, Hannah Jongebloed, Brenton Baguley, Skye Marshall, Victoria M White, Patricia M Livingston, Kathy Bell, Leonie Young, Sabe Sabesan, Dayna Swiatek, Anna Boltong, Joanne M Britto, Anna Ugalde

**Affiliations:** Institute for Physical Activity and Nutrition, Deakin University, Geelong, VIC, Australia; Department of Health Services Research, Peter MacCallum Cancer Centre, Melbourne, VIC, Australia; Institute for Health Transformation, Deakin University, Geelong, VIC, Australia; Institute for Physical Activity and Nutrition, Deakin University, Geelong, VIC, Australia; Institute for Health Transformation, Deakin University, Geelong, VIC, Australia; Cancer and Palliative Care Outcomes Centre, School of Nursing, Queensland University of Technology, Brisbane, QLD, Australia; Bond University Nutrition & Dietetics Research Group, Faculty of Health Sciences and Medicine, Bond University, Gold Coast, QLD, Australia; School of Psychology, Faculty of Health, Deakin University, Geelong, VIC, Australia; Institute for Health Transformation, Deakin University, Geelong, VIC, Australia; Faculty of Health, Deakin University, Geelong, VIC, Australia; Clinical Oncology Society of Australia, Sydney, NSW, Australia; Clinical Oncology Society of Australia, Sydney, NSW, Australia; Clinical Oncology Society of Australia, Sydney, NSW, Australia; Department of Medical Oncology, Townsville Cancer Centre, Townsville, QLD, Australia; Faculty of Health, Deakin University, Geelong, VIC, Australia; Kirby Institute, University of New South Wales, NSW 2052, Australia; Department of Nutrition, Dietetics and Food, Faculty of Medicine, Nursing and Health Sciences, Monash University, Clayton 3800, Victoria, Australia; Victorian Comprehensive Cancer Centre Alliance, Parkville, VIC, Australia; Institute for Health Transformation, Deakin University, Geelong, VIC, Australia

## Abstract

**Objective:**

Although the benefits of consumer involvement in research and health care initiatives are known, there is a need to optimize this for all people with cancer. This systematic review aimed to synthesize and evaluate the application of co-design in the oncology literature and develop recommendations to guide the application of optimal co-design processes and reporting in oncology research, practice, and policy.

**Methods:**

A systematic review of co-design studies in adults with cancer was conducted, searching MEDLINE, CINAHL, Embase, and PsycINFO databases and included studies focused on 2 concepts, co-design and oncology.

**Results:**

A total of 5652 titles and abstracts were screened, resulting in 66 eligible publications reporting on 51 unique studies. Four frameworks were applied to describe the co-design initiatives. Most co-design initiatives were designed for use in an outpatient setting (n = 38; 74%) and were predominantly digital resources (n = 14; 27%) or apps (n = 12; 23%). Most studies (n = 25; 49%) used a co-production approach to consumer engagement. Although some studies presented strong co-design methodology, most (n = 36; 70%) did not report the co-design approach, and 14% used no framework. Reporting was poor for the participant level of involvement, the frequency, and time commitment of co-design sessions. Consumer participation level was predominantly collaborate (n = 25; 49%).

**Conclusions:**

There are opportunities to improve the application of co-design in oncology research. This review has generated recommendations to guide 1) methodology and frameworks, 2) recruitment and engagement of co-design participants, and 3) evaluation of the co-design process. These recommendations can help drive appropriate, meaningful, and equitable co-design, leading to better cancer research and care.

Patient and public involvement is critical in both healthcare delivery and health research (1). This importance is recognized by national research funding bodies in the United Kingdom (2), the United States (3), and Australia (4), among other countries, whereby evidence of consumer involvement in research programs has become a key expectation. Toolkits have been developed to guide consumer engagement and establish formal pathways for consumer involvement (5-7). These recommendations place the consumer voice at the center of health improvement initiatives to maximize relevance and effectiveness in improving outcomes.

Participatory action research is an overarching term used to encompass consumer involvement in healthcare research and services (1,8). Co-design is one participatory action research approach to consumer representation that has been increasingly used over the past decade. Co-design is defined as a collaboration between researchers and stakeholders, including people affected by a health condition, healthcare services, health professionals, policy makers, and other interested parties to actively develop an intervention, service, or product (9,10). Various co-design methodologies are described in the literature (11,12); however, a common underlying theme is an iterative process of coming together to generate and refine ideas over time to deliver a solution to a problem (11,12).

There are examples of how co-design should be conducted. For example, a systematic review (13) and stakeholder consultation (14) informed a set of key principles and best practices for co-design in health with First Nations Australians. As oncology research progresses, there is a need for interventions, resources, and programs to adopt best practice co-design, and there is opportunity for a thorough exploration of co-design approaches, examining factors such as strategies used, activities undertaken, level of participation of stakeholders, and components of the co-design process. This examination will identify strengths and areas requiring improvement with the current co-design literature, advance knowledge of optimal co-design processes, and inform recommendations for improvement.

The aims of this review were to: 1) synthesize and evaluate the application of co-design in the oncology literature involving adults affected by cancer, and 2) form recommendations to guide the application of optimal co-design processes and reporting in oncology research, clinical practice, and policy.

## Methods

This systematic review complies with the PRISMA (Preferred Reporting Items for Systematic reviews and Meta-Analyses) (15) statement and was registered with the International Prospective Register of Systematic Reviews (PROSPERO CRD42023404965). A second phase of the review was to generate recommendations.

### Search strategy

A systematic search was conducted across the following databases on the March 6, 2023: MEDLINE Complete, CINAHL Complete, Embase, and PsycINFO. The search strategy (Supplementary Table 1, available online) was developed with the assistance of a medical librarian and focused on two concepts, co-design and oncology, and included both indexed headings and free terms. The search was limited to peer-reviewed articles published in English, and no date limiters were applied. The reference lists of included articles were reviewed for additional publications meeting eligibility requirements. Forward citation searching on the included articles was undertaken to identify additional articles by the same author group that reported further components of the same co-design initiative, for example, qualitative interviews, as well as to identify evaluation and implementation of the co-designed initiatives.

### Selection criteria

Papers were eligible for inclusion if they met the following criteria: 1) involved adults (18 years or older) diagnosed with cancer as participants in the co-design process, and 2) the co-design process had been applied to the development or adaptation of any patient-facing intervention, program, or resource with a primary focus on cancer care. Despite the popularity of using co-design in healthcare, a universally agreed definition of the term is not available. For the purpose of this review and to capture the range of literature on this topic, we adopted a broad concept of co-design used as an overarching term to refer to an active engagement between stakeholders in the design of a product. In this review, “consumer” refers to people with a current or previous diagnosis of cancer, whereas “stakeholder” refers to people with cancer, carers, health professionals, and any other type of stakeholder. All original study designs including quantitative, qualitative, and mixed-methods studies were eligible. Papers were considered ineligible if they 1) focused on an evaluation of an existing intervention, program, or resource instead of co-design, 2) were aimed at people receiving palliative care, 3) the initiative was not designed exclusively for cancer care, 4) did not include people with cancer in the co-design process or included any participant under the age of 18. Reviews, conference abstracts, letters to the editor, and protocols were ineligible. The gray literature was not included in this review.

### Study selection

Four authors (NK, HJ, BB, AU) completed the title and abstract and full text screening in a blinded, standardized manner using Covidence systematic review software (Veritas Health Innovation, Melbourne, Australia). All four authors completed both the initial title and abstract screening and full text review to determine eligibility for inclusion. Conflicts were resolved through discussion until consensus was reached.

### Data extraction

**Figure 1.**
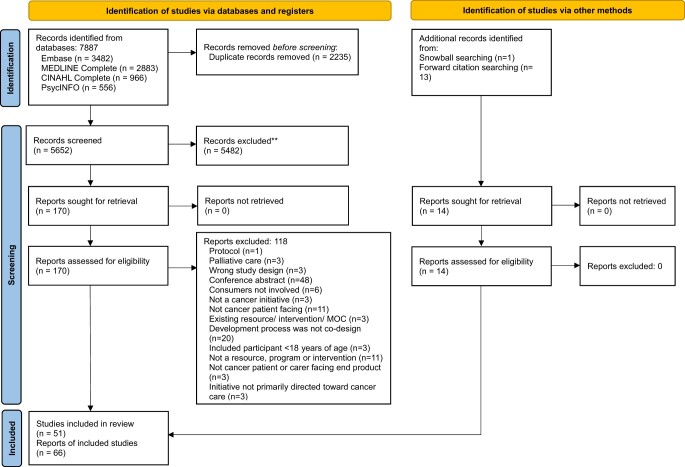
PRISMA flow diagram.

Microsoft Excel software was used to complete data extraction. The data extraction tool captured the following from the included studies: 1) country, 2) target setting and group for the co-design initiative, 3) alignment between the target group and co-design participants with cancer, 4) description and purpose of the co-design initiative, 5) initiative stage (development or adaptation), and 6) evidence of evaluation or implementation of the co-design initiative. In addition, four frameworks were applied to analyze the co-design initiative for 1) co-design consumer engagement approach (1), 2) details of the co-design process (16), 3) stakeholder participation across the full co-design process (9), and 4) consumer participation level according to power held by consumers (17) (Figure 1). Stakeholders were considered any participant in the co-design process excluding the research team and external service providers and were categorized according to type: consumer (people with a current or previous diagnosis of cancer), carer, health professional, or other. As consumer participation levels varied depending on the project stage, the highest level achieved during the study by a consumer was selected for coding.

### Recommendations

A co-design approach was used to generate recommendations based on the findings of the review. At the commencement of the review, an expert group of stakeholders in consumer engagement in cancer care were selected as authors. The stakeholder authors include consumers with lived experience of cancer, senior representatives from key oncology and government organizations in Australia who have led national initiatives on consumer participation in health care, and researchers who work closely with consumers within their research programs. The stakeholder authors were involved in the development of the review protocol and in the generation of the recommendations using the following methodology: 1) a draft list of recommendations were generated from the findings by the lead author, 2) a 60-minute workshop was held, led by the lead author and consumer stakeholders, to present and explore the results and discuss the draft recommendations, 3) comments and feedback from the workshop were incorporated and organized into themes to refine and revise the draft recommendations by the lead author following the workshop, 4) the revised thematic recommendations were distributed to the stakeholder authors in two rounds of consultation, thus producing the final recommendations reported. During the revision of each draft of recommendations, the following were considered: reflection of the alignment with findings of the review, reflection of alignment with guidelines for consumer engagement (4,6), and ensuring the representation of the consumer authors.

The data extraction tool was piloted and refined by three authors (AU, HJ, SM). Data extraction and study coding according to the 4 frameworks were undertaken by one author (SM) due to the complexities and importance of consistency. During this process, SM met regularly with 2 other authors (NK, HJ) to review and confirm data extraction and discuss any discrepancies or issues arising until concordance was achieved.

### Data synthesis and analysis

A narrative synthesis of findings was conducted using the SWiM (Synthesis Without Meta-Analysis) guidelines (18). Descriptive statistics were calculated using Microsoft Excel to summarize included studies. Key characteristics of the included studies were presented in tabulated format with additional information provided to describe the allocation of studies to the characteristic groups. To examine the frameworks or methodological approaches underpinning the co-design process, studies were broadly grouped into those using a formal methodology specifically for co-creation, co-design, or co-production, those based on general principles for user involvement, those based on frameworks to guide design or intervention development, those using published standards for development of resources, those using other broad research methodologies, and those not using any framework. Descriptive statistics were used to determine the proportion of initiatives meeting the description for each of the elements of co-design by Boyd et al. (9) and the consumer participation levels described in the Agency for Clinical Innovation (17).

## Results

**Figure 2.**
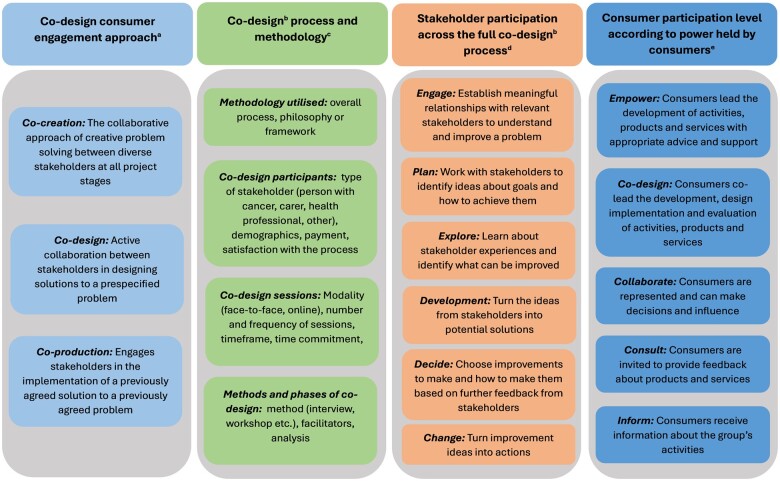
Description of the frameworks applied to analyze the co-design initiatives. ^a^Vargas et al. (1); ^b^co-design used as the overarching term; ^c^adapted from a systematic review by Eyles et al. (16); ^d^Boyd et al. (9); ^e^Agency for Clinical Innovation, page 6 (17). Consumer refers to people with cancer. Stakeholder refers to all codesign participants including people with cancer, carers, health professionals, or other stakeholders.

A total of 7887 records were identified from the electronic databases from which 2235 duplicates were removed, leaving 5652 title and abstracts that were screened (Figure 2). After title and abstract screening, 170 publications were retained and assessed for eligibility. Handsearching the reference lists of included articles identified 1 additional study, and in the process of data extraction an additional 13 papers were identified that reported further details of an included co-design initiative, resulting in 66 eligible publications reporting on 51 unique studies. Throughout the results, the term co-design is used as an overarching term to describe the broad initiative, unless referring to the Vargas definition of co-production, co-design, or co-creation as a description of the consumer engagement type.

### Overview of included studies and co-design initiatives

A summary of the characteristics of each study is described in Table 1, and an overview of each included study is presented in Supplementary Table 2 (available online) (19-84). The most common country for the conduct of studies was the United States (n = 14; 27%), and the majority of initiatives were designed for use in an outpatient setting (n = 38; 74%). Almost half of the studies (n = 25; 49%) described initiatives intended for people with any cancer diagnosis and their carers, with breast cancer (n = 12; 23%) the second most common group. The predominant type of co-design initiatives included digital resources (n = 14; 27%) or apps with or without supporting resources (n = 12; 23%), and most initiatives were developing a new initiative (n = 39; 76%). The duration of the co-design process ranged from 2 months up to 60 months, and more than half (n = 29, 57%) were 6 months or longer; however, in 14 studies no duration was reported. The publication of co-design initiatives has increased over time, with 5 (10%) studies published before 2015, 13 (25%) between 2015 and 2019, and 33 (65%) from 2020 onward. However, many publications reported on studies conducted many years earlier.

**Table 1.** Characteristics of included studies (N = 51)

Characteristic	No. of studies (%)
Country	
United States	14 (27)
Netherlands	11 (21)
Australia	10 (20)
United Kingdom	9 (18)
Other	7 (14)
Consumer stakeholder engagement type^a^	
Co-production	25 (49)
Co-design	15 (29)
Co-creation	11 (22)
Target community for co-design initiative^b^	
Outpatient	38 (74)
Community	9 (18)
Online	3 (6)
Mixed (inpatient and outpatient)	1 (2)
Cancer type addressed by the co-design initiative	
Any cancer diagnosis	25 (49)
Breast cancer only	12 (23)
Prostate cancer only	6 (12)
Other specific cancer diagnoses	8 (16)
Initiative type	
Digital resource^c^	14 (27)
App with or without other resources	12 (23)
Patient decision aid	7 (14)
Non-digital intervention or program^d^	7 (14)
Digital intervention or program^e^	6 (12)
Nondigital resource^f^	4 (8)
Serious game	1 (2)
Initiative stage	
Development of new initiative	39 (76)
Adaptation of existing initiative	11 (22)
Combination	1 (2)

a Definition from Vargas et al. (1).

b Refers to how the target community were engaging with the health service while they were using the initiative; for example, a digital resource that was used with people attending as outpatients or a digital resource that was used by people within an online support group.

c Digital resources included initiatives that were intended to provide information and were accessible in an online format but did not directly provide health care, support, or intervention, for example, an informational website.

d Nondigital interventions or programs were those delivered by a health professional without a digital or virtual platform such as in person or via telephone, for example, a cognitive behavioral therapy program.

e Digital interventions or programs were those delivered health care, support, or interventions using an online format, for example, an interactive website.

f Nondigital resources included initiatives that were intended to provide information and were provided in a nondigital format, for example, an informational booklet.

### Co-design consumer engagement approach

In seven (14%) studies, the authors’ description of their process as co-design, co-production, or co-creation aligned with the definitions by Vargas et al. (1), and in eight (16%), the authors’ definition was misaligned, with 50% at a lower level of consumer engagement; however, in most studies (n = 36, 70%), no definition was reported. Most studies (n = 25; 49%) used a co-production approach to consumer engagement. There were regional differences in the use of co-design, co-production, and co-creation approaches, with North American and European studies predominantly using co-production (60% and 66%, respectively), and Australian studies predominantly using a co-creation approach (50%), whereas in the United Kingdom the three approaches were used evenly.

### Co-design process and methodology

The methodology, philosophy, or framework underpinning the overall co-design process across the studies are presented in Table 2 as: 1) formal co-design, co-production, or co-creation methodologies; 2) general principles for user involvement; 3) design or intervention development frameworks; 4) standards for resource development; 5) other approaches; and 6) no framework (Table 2). The most widely used methodologies were user-centered design (21%) and experienced-based co-design (10%), whereas 14% of studies reported no framework or methodology.

**Table 2.** Co-design framework or approach used by included studies (N = 51)

Co-design^a^ approach	No. of studies^b^ (%)
Formal co-design, co-production, or co-creation methodology (23,42,48-52,57,73)	
Experience-based co-design	5 (10)
Design council’s double diamond model	1 (2)
Framework for the co-production and prototyping of public health interventions	1 (2)
General principals for user involvement (19-22,24,25,27,29-31,34,36-39,43-45,53,54,60-62,65-68,74,75,77,78,81,82)	
Participatory design approach	3 (6)
Community-based participatory research	1 (2)
User-centered design	11 (21)
Human-centered participatory design	3 (6)
National institute for health and care research national standards for public involvement in research	1 (2)
Participatory action research	3 (6)
Interactive learning and action approach	1 (2)
Participatory health approach	1 (2)
Person-based approach to intervention development	2 (4)
Action research	1 (2)
User driven co-design	1 (2)
Design or intervention development framework (26,28,32,33,44,47,51,55,56,65,72,77,83)	
Design thinking research process	1 (2)
Agile framework	3 (6)
mHealth development and evaluation framework	1 (2)
Medical research council framework for developing and evaluating complex interventions	4 (8)
Framework for the co-production and prototyping of public health interventions	1 (2)
Centre for ehealth research and disease management comprehensive roadmap	1 (2)
Scrum framework	2 (4)
Spiral technology action research	1 (2)
Standards for resource development (20)	
International patient decision aid standards	1 (2)
Other approaches (20,69,70)	
Phenomenological qualitative study	1 (2)
Mixed methods	1 (2)
No framework or approach (35,58,59,63,64,71)	7 (14)

a Co-design used as the overarching term to describe the broad initiative.

b Multiple approaches used by some studies.

The majority of studies (96%) used multiple methods, eg, focus groups, workshops, and user testing to co-design the initiative. Different methods were often (n = 40; 78%) used for different stakeholder types. The most common methods used with consumers included interviews (n = 37; 73%), user testing (n = 25; 49%), focus groups (n = 19; 37%), workshops (n = 17; 33%), and consultation (n = 15; 29%). Health professionals or other stakeholders were included in 47 (92%) of the 51 studies. Interviews (49%) and consultation via steering committees, advisory panels, or email (36%) were the most common methods used with health professionals and other stakeholders. Carers were included as stakeholders in a third of the studies (n = 17; 33%), and they were most likely to be involved in focus groups (41%), workshops (35%), or interviews (35%). The modality of co-design engagement (face-to-face, online, phone or videoconference) was poorly reported, with 28 (55%) studies not reporting the modality for at least one the methods used. Where modality was reported, face-to-face was by far the most used for interviews, focus groups, workshops, and user testing, whereas online in the form of email was most used for consultation.

The number of co-design sessions ranged from 1 to 17 for consumers (≥4 in n = 30; 59% studies), and 1 to 12 for carers, healthcare professionals, or other stakeholders. The frequency of sessions was not reported, partially reported, or unclear in 32 (61%) of the 51 studies, and where this was reported, most were one-off interviews, focus groups, or workshops (n = 11; 21%). The time commitment for stakeholder involvement in the co-design process was similarly poorly reported with 35 (69%) of 51 studies not reporting or partially reporting this information. Where the time commitment was reported, this ranged from 8 minutes to 3 hours for interviews (reported in 24 studies), 1 to 6 hours across focus groups (reported in 10 studies), 2 to 6 hours for workshops (reported in seven studies), and 10 minutes to an unspecified amount of time over a 6-week period for user testing (reported in 13 studies).

The co-design sessions were facilitated by the research or project team in 47 (92%) studies. In the remaining 4 studies, clinicians, external representatives, or independent facilitators were used. Analysis of the information gained from the co-design activities varied depending on the type of activity. Interviews and focus groups were predominantly subject to thematic or content analysis (32 [86%] of 37 studies; 11 [58%] of 19 studies, respectively). Feedback from consultations was summarized, but most often the method for analysis was not reported. User testing was typically analyzed using descriptive statistics or subject to thematic analysis from think aloud or qualitative interviews. The approach to analysis of workshops was diverse, employing 12 different techniques, some of which included thematic or content analysis, polling, concept mapping, and discussions to prioritize and summarize content.

Stakeholder satisfaction with the co-design process or experience was evaluated in only 3 studies using reflective feedback (23,49,50) or an evaluation questionnaire (24,25).

### Co-design stakeholders

Reported sample sizes for stakeholders ranged from 11 to greater than or equal to 220. Many studies failed to report the number of stakeholder participants for one or more co-design methods; therefore, the extracted sample sizes reflect the minimum plausible number of stakeholders. For example, a study that described the role and function of a patient advisory group but did not describe the group members was coded as having greater than or equal to 2 participants. Sample sizes ranged from 2 to greater than or equal to 150 for consumers, 1 to 75 for healthcare professionals, 1 to 30 for carers, and 2 to greater than or equal to 26 for other stakeholders. The remainder of this section will focus on the consumer stakeholders.

Excluding sex-specific cancers, females represented a median of 61.5% of the samples; (range: 29% to 82%; sex not reported in six studies). The age range of co-design participants with cancer varied widely, with the youngest participants aged 21 years, and the oldest participant aged 91 years. Age was not reported in 11 studies.

Consumers were iteratively engaged, ie, the same consumers were engaged in each step of the co-design process in 48 (94%) studies. The most common techniques to recruit co-design participants with cancer were convenience sampling (n = 27, 53%) (19,21,22,24-26,28,30,32,35,36,43,51-57,60,61,63,64,68,69,71,74-76,78,79,81,82) or a mix of convenience and purposive sampling (n = 9, 18%) (23,27,33,38,40-42,45,47,49,50,67,72,80,84). In 7 studies, an attempt was made to achieve diversity in demographic characteristics such as age, education level, and sex (20,24,25,28,38,67,72,77), and 3 studies specifically targeted people with a range of experiences, health literacy levels, and information needs (27,42,45,49,50). Five studies acknowledged the lack of diversity among their co-design participants with cancer (23,44,64,65,69,81), and 4 studies reported targeting specific minority or under-represented ethnicities, race, or geographical areas (21,30,73,83). However, in the majority of studies (63%), no statement regarding diversity and equity was included. In most studies (n = 31, 61%), there was alignment between the target group for the co-design initiative and the consumer participants, and in 10 (20%) studies, there was partial alignment. Where there was no alignment, this was generally due to consumer participants not representing the stage of the treatment the co-design initiative was targeting, for example, cancer survivors 1 to 2 years post-treatment co-designing an initiative regarding cancer treatment. Payment of consumer stakeholders was described in 13 (25%) studies (22,32,35,50,58,59,61,64,67,72,74,77,81), of which 11 reported payment for participation in the co-design process, and 2 reported reimbursement of travel costs or expenses related to participation. For the studies that included payment, payment was provided for only some components of consumer participation, for example, workshop but not survey participation. Of the studies reporting consumer payment, most (7 of 13) used a co-production approach to consumer engagement.

### Stakeholder participation across the full co-design process

The most common elements of co-design within the studies were *development* (turn ideas from stakeholders into solutions), which was used in 98% (n = 50) of studies, *explore* (learn about stakeholder experiences and what can be improved), which was used in 94% (n = 48) of studies, and *engage* (establish meaningful relationships with stakeholders), which was used in 90% (n = 46) of studies (Table 3). *Plan* (working with stakeholders to identify and achieve goals) was the least used element of co-design, used in 57% (n = 29) of studies. Only 15 (29%) studies addressed all six elements of co-design with 1 or more of the stakeholder groups. Consumers were the participant group most commonly included in each element of co-design (53%-94%), followed by healthcare professionals (29%-75%), whereas carers were the least included group (8%-24%).

**Table 3.** Proportion of included studies that addressed each of the six the elements of co-design with any participant or specific stakeholder groups

Co-design^a^ element	Any participants N (%)	Consumers N (%)	Carers N (%)	Healthcare professionals N (%)	Other stakeholders N (%)	Summary of methods used for each co-design element
Engage^b^	46 (90)	44 (86)	11 (22)	38 (75)	12 (24)	Steering committee meetings, advisory boards, expert panels, interviews, workshops, focus groups, surveys, consultation
Plan^c^	29 (57)	27 (53)	4 (8)	19 (37)	10 (20)	Workshops, stakeholder advisory groups, literature review, small group discussion, interviews, surveys, knowledge cafes, consultation
Explore^d^	48 (94)	48 (94)	8 (16)	32 (63)	8 (16)	Focus groups, interviews, stakeholder advisory groups, needs assessment, usability testing or surveys, workshops, knowledge cafes, field observation
Development^e^	50 (98)	48 (94)	12 (24)	37 (73)	14 (27)	Workshops, interviews, focus groups, stakeholder advisory groups, usability testing, consultation
Decide^f^	38 (75)	32 (63)	8 (16)	24 (47)	11 (22)	Workshops, usability testing, group and individual meetings, consultation, interviews, consensus conference, expert panels
Change^g^	37 (73)	35 (69)	6 (12)	15 (29)	9 (18)	Usability testing, feasibility studies, interviews, workshops, consultation

a Co-design used as the overarching term to describe the broad initiative.

b Establish meaningful relationships with consumers and/or relevant stakeholders to understand and improve a problem.

c Work with consumers and/or stakeholders to identify ideas about goals and how to achieve them.

d Learn about consumers and/or stakeholders experiences and identify what can be improved.

e Turn the ideas from consumers and/or stakeholders into potential solutions.

f Choose improvements to make and how to make them based on further feedback from consumers and/or stakeholders.

g Turn improvement ideas into actions (9).

### Power held by consumers

According to definitions by the Agency for Clinical Innovation (17), the level of power held by consumers in the co-design process was making and influencing decisions *(collaborate)* in 25 (49%) studies, co-leading development, design, implementation, and evaluation of the initiative *(co-design)* in 19 (37%) studies, and providing feedback about products and services developed *(consult)* in seven (14%) studies. The criteria for the highest and lowest level of consumer power, leading development of activities, products and services *(empower)*, and receiving information about the initiatives activities *(inform)*, were not met by any of the studies.

### Efficacy evaluation and implementation

In 16 (31%) studies, the authors had published an evaluation of the co-designed initiative or reported an evaluation of the initiative within the same co-design publication. A higher proportion (n = 25, 49%) reported planned future work, which included pilot feasibility studies, randomized trials to evaluate efficacy, implementation trials, or further adaptation or development of the co-design initiative. Evidence of implementation or rollout of the co-designed initiative was reported in 20 (39%) studies, in the form of local integration into usual care, online availability of the resource produced by the initiative, or via app stores.

### Recommendations

**Figure 3.**
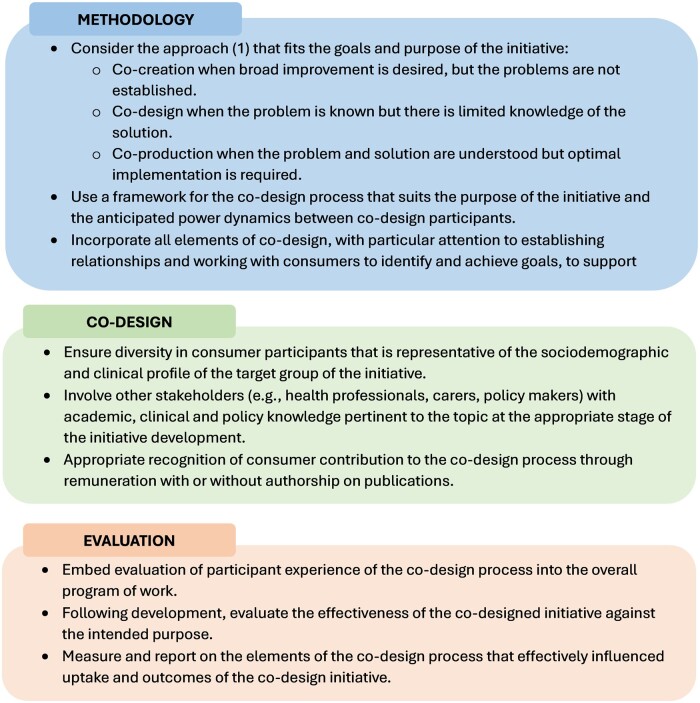
Recommendations for researchers, clinicians and consumer advocacy organizations for the application of co-design in oncology. Co-design used as an overarching term to describe the broad initiative, whereas co-design approach is as defined by Vargas et al. (1).

Themes generated from the workshop and consultation process with stakeholder authors were 1) methodological issues, including inconsistent reporting, use of terminology and frameworks specific to co-design, no clear rationale for the choice of a co-production, co-design, or co-creation approach to consumer engagement, limited consideration of power dynamics between consumers and other stakeholders, and inconsistent engagement of consumers iteratively throughout the entire process; 2) recruitment and selection of co-design participants where there was limited justification for the choice or diversity of consumer and other stakeholder participants, how the types of consumers and stakeholders recruited supported the purpose of the co-design initiative, and limited reporting of how consumers were recognized via reimbursement or authorship; 3) evaluation where there was poor reporting of both evaluation of the co-design process and the co-designed initiative limiting any assessment of factors that support the effectiveness or uptake of co-designed initiatives. Based on these themes, recommendations relating to (1) co-design methodology, (2) recruitment and engagement of consumer and other stakeholder participants, and (3) evaluation of co-design initiatives were generated (Figure 3).

## Discussion

This systematic review synthesized and appraised the current literature on the application of co-design as a broad concept of active engagement between stakeholders in the design of a product in the oncology literature. There are inconsistencies with reporting regarding the approach used for consumer engagement, methodologies underpinning the co-design process, and framework used to guide the process. There was limited reporting of components of the co-design process, including the number and sociodemographic profile of stakeholders, frequency of co-design sessions, the time commitment involved, and whether consumer participants were remunerated. Despite most studies involving consumers who clinically represented the target group of the co-design initiative, there was a lack of consideration of diversity and equity. A minority of studies encompassed all 6 elements of co-design with one of more of the stakeholder groups. Overall, there are several opportunities to improve co-design in oncology research.

Given the gaps in co-design processes and reporting, we suggest key recommendations (Figure 3) to guide the optimal application and reporting of co-design methodology to inform interventions, resources, and services for people with cancer. Our systematic review highlighted inconsistencies in reporting and a lack of clear application of definitions across studies involving co-design initiatives in oncology. Clear, accurate, and reliable reporting of research methodologies is now a mandatory requirement of many peer-reviewed journals to enhance transparency and consistency across research studies. This has led to the development of reporting guidelines relevant to a multitude of research designs and methodologies (85). At present, there is no reporting guideline available for co-design methodologies, which may relate to the relative recency of growth in the popularity of publishing co-design methods, as observed in our review where more than two-thirds of the included studies were published from 2020 onward. The Guidance for Reporting Involvement of Patients and Public (GRIPP) checklists 1 and 2 were published in 2011 (86) and 2017 (87), respectively, based on systematic reviews of the evidence base in patient and public involvement (88,89). However, the GRIPP checklist is designed for patient and public involvement in research generally and is not specific to consumer or other stakeholder involvement in co-design initiatives.

For this review, we applied the definitions proposed by Vargas et al. to distinguish between studies that had used a co-creation, co-design, and co-production approach to consumer engagement (1) for which there are nuances in for what purpose consumers and other stakeholder are engaged. The vast majority of studies included in our review had either incorrectly described the consumer engagement approach used or had not described it at all; however, we acknowledge the historical lack of clarity for these terms until the publication of the Vargas definitions in 2022. This is important from a scientific perspective for an accurate description of the approach and to enable comparison of outcomes from the development of initiatives across studies using the same approach. More important is that people undertaking co-design appropriately choose the approach for the planned initiative, which should be guided by the purpose of the initiative. Co-creation involves problem solving at all stages of the project where the problem is not yet defined and the outcome is not established (1). Co-creation may be best suited to circumstances where the intention is broad improvements although the underlying problems are not established, and consumers and other stakeholders need to be engaged to develop an understanding of and inform the issues. In contrast, co-design may be best used when the problem has been identified and the purpose of engaging consumers and other stakeholders is to collaboratively design the solution (1). Co-design may be most appropriate when significant effort has already been invested in understanding the problem although knowledge to support a solution is limited. Co-production involves an already agreed problem and solution (1), meaning it may be most appropriate when the problem is well understood and evidence is available to underpin the solution or the solution is co-designed with other stakeholder groups such as healthcare professionals, and consumers and other stakeholders are engaged to optimize implementation.

Several frameworks to guide co-design processes are available, with examples including Experience-Based Co-Design and the British Design Council’s Double Diamond frameworks (90,91). These frameworks provide structure around the steps to be followed in a co-design process regardless of the underlying methodology used. Greenhalgh et al. published a systematic review of published frameworks that supported patient and public involvement in research (92). The authors identified 65 different frameworks that were subsequently grouped into categories based on the intended purpose; these included power-focused, priority-setting, study-focused, report-focused, and partnership-focused (92). Although these frameworks were not specifically intended for co-design, they provide principles and structure to the engagement of consumers in the co-design process. In addition, a robust framework has recently been established after publication of a set of key principles and best practices for co-design of health initiatives with First Nations Australians (14). A wide variety of frameworks or standards were used by the studies included in our review, ranging from those designed specifically for co-design or intervention development to general principles for user involvement such as user-centered design. Similar to the need to choose the right approach of co-creation, co-design, and co-production, consideration is required regarding the most appropriate framework that is grounded in the overall purpose of the initiative and the anticipated power dynamics among co-design participants.

This review has uncovered specific challenges related to the selection and recruitment of consumers as co-design participants. Few studies specifically reported an effort to ensure diversity in their sample or to address the needs of minority groups, including reporting of Indigenous or First Nations status. Diversity has been described as an asset and an essential component of co-design (93). The lack of diversity evident in this review may reflect the predominant use of convenience sampling by most studies, where in some studies consumers were selected due to pre-existing relationships with the research team rather than their representation of the target group. It is acknowledged that consumer representatives tend to be from a higher socioeconomic demographic, meaning a dedicated attempt to achieve diversity is necessary (94). One study in which this was done well had surveyed or interviewed patients and carers in the first stage of an experience-based co-design initiative and drew on respondents for the subsequent phase involving workshops, where purposive sampling was used to ensure diversity in patient and carer experiences, cancer diagnosis, age, sex, and geographical location (50). Co-design initiatives where the target group were people newly diagnosed with cancer presented a unique challenge since recruitment of the target group as consumer participants may not be appropriate due to the significant physical and psychological burden of this period. In these cases, people who had completed treatment for the same diagnosis were recruited instead (62,63,73). In one study, the target group was particularly vulnerable, involving older migrant adults with cancer, and was deemed unable to contribute to the development, decision making, and co-leadership phases of the co-design process (72). The description of the recruitment and selection of consumers and other stakeholders was not generally well reported. Selection and recruitment of consumers and other stakeholders are further components that require forethought and planning to ensure representativeness and iterative engagement of the target group of people with cancer with lived experience alongside involvement of people with academic, clinical, or policy knowledge pertinent to the topic at each stage of the initiative.

Few studies encompassed all elements of co-design, limiting opportunities for early and genuinely iterative involvement of consumers across the entire co-design process. *Plan*, which represents working with consumers and other stakeholders to brainstorm the goals for the co-design initiative and how these could be achieved (9), was the element least used by the included studies despite being integral to informing the direction of the initiative. This aligns with the predominance of studies using a co-production approach in which there is no consumer input into a problem or solution. In the included studies, what represented consumer involvement across the stages of the project varied widely. This was particularly evident in *Development,* which had the greatest divergence in how consumers and other stakeholders contributed, although it was one of the most used elements of co-design. This divergence was particularly apparent in in the decision-making power of consumers and other stakeholders during *Development.* Decision making ranged from relatively passive, where consumer and other stakeholders’ ideas were sought although researchers decided if the idea was used and development of the initiative occurred with no or minimal further input from consumers or other, for example, researchers using data from surveys and interviews with stakeholders (45), to more active, where co-design participants had an equal say in whether an idea progressed and actively contributed to initiative development, for example, the design of prototypes during workshops (42,50). Studies using co-creation or co-design workshops or those including consumer and other stakeholders as co-researchers tended to lean more toward more active decision-making power for the consumers and other stakeholders, whereas those using surveys, interviews, or focus groups alone leaned toward relatively passive decision-making power. The level of engagement of consumers in the co-design process appeared to be unrelated to the number of co-design participants, with some studies having a small number of participants with cancer, albeit with highly active involvement as investigators.

To strengthen our understanding of what constitutes effective co-design, co-design initiatives must be evaluated using a multipronged approach. First, an evaluation of the experience of the co-design process from the consumer viewpoint is critical to improving our knowledge of effective consumer engagement. Our review revealed only 3 studies had undertaken such an evaluation, demonstrating a missed opportunity for knowledge gain. In addition, substantial time and resources are invested in co-design initiatives, with the assumption that such processes will increase the likelihood of implementation into practice as well as uptake by the target group. However, this requires a robust evaluation of the co-designed initiative. Of the studies included in this review, 31% had completed an evaluation, and in 39% there was evidence of rollout or implementation of the initiative, although in some instances an evaluation may not yet be published. Increasing the completion rate and dissemination of evaluation results could act to advance our understanding of the effectiveness of co-design in oncology, and authors should consider including any evidence, for example, gray literature or social impact, regarding effectiveness of their co-designed initiative.

A strength of this review is the use of frameworks that enabled extensive evaluation of the level of engagement of co-design participants, methodologies, and approaches underpinning the co-design process in a large number of studies to generate recommendations for the optimal application and reporting of co-design initiatives. The identification of additional articles during data extraction consolidated co-design initiatives where different phases were reported across multiple publications. However, this review has some limitations. Although databases were extensively searched, a wide range of search terms was used, and a large number of studies were included in this review, initiatives not clearly defined as co-design or a related term may have been excluded. The description of consumer and other stakeholder participants relied on the reporting of the included studies, and in the case of non-consumer stakeholders, it was often difficult to differentiate between healthcare professionals or other stakeholders. We did not record whether consumer participants co-authored publications because this was challenging to detect, although this would have been an interesting inclusion. This review focused on published co-design initiatives, and we acknowledge this may have excluded co-design initiatives that were not publicly reported. In addition, we were unable to determine what features of co-design initiatives lead to improved patient outcomes or uptake of the initiative into practice since this relied on the reporting of further trials or implementation studies beyond the co-design process. This information was sought through forward citation searching of the included articles; however, most of the included studies were published from 2020 onward and evaluation or implementation trials may not yet be published. To achieve consistency within complex data, the coding of included studies to the co-design frameworks was conducted by a single review investigator and therefore may not align with judgments of others. Many included studies were reported poorly, particularly regarding stakeholder characteristics and engagement. This meant some extracted data reflected the interpretations of study methods by the review investigator; therefore, some extracted data may not be an accurate reflection of the study, further emphasizing the requirement for reporting guidelines to improve clarity of future co-design studies.

## Conclusions and future directions

Co-design is an important and increasingly used methodology for engaging stakeholders in the development of interventions, resources, and programs for people with cancer. This systematic review demonstrated there are opportunities to improve the application of co-design in oncology research through accurate reporting and description of the co-design process and participants; appropriate choice of methodology and frameworks to gain meaningful input by consumers; and thoughtful consideration of the composition of consumer and other stakeholder participants to reflect the target group of the initiative and the expertise required during development. This review has generated recommendations to guide researchers, clinicians, health services, government, policy makers, and consumer advocacy organizations in leading robust co-design processes. Future work is required to develop reporting guidelines for co-design studies, qualitatively explore participants’ experience of the co-design process, and evaluate co-design studies to increase our understanding of whether co-design results in end products that better meet the needs of people with cancer and ultimately lead to improved uptake of interventions as well as clinical and health outcomes.

## Supplementary Material

pkae048_Supplementary_Data

## Data Availability

This is a systematic review and all data included in this paper are from publicly available research papers.
