# Supplementary material for: Meaningful consumer involvement in cancer care: a systematic review on co-design methods and processes
Source: JNCI Cancer Spectr. 2024 Jun 19;8(4):pkae048. doi: 10.1093/jncics/pkae048 (PMC11240760; doi:10.1093/jncics/pkae048)
Supplement: pkae048_Supplementary_Data [file pkae048_supplementary_data.pdf]

Supplementary Table 1: Search terms

	Concept 1 – Co-design	Concept 2 - Cancer
MeSH Headings	Medline - Community-Based Participatory Research	Medline – Neoplasms, Medical Oncology<br>CINAHL – Neoplasms, Oncology<br>PsycInfo - Neoplasms, Oncology
Emtree Terms	'user-centered design', 'patient engagement', 'stakeholder engagement', 'product design', 'participatory action research', 'participatory research'	'cancer diagnosis', 'cancer rehabilitation', ‘malignant neoplasm’, ‘carcinoma’, ‘oncology’, 'neoplasm'
Free Terms	co-design*, codesign*<br>co-creat*, cocreat*<br>co-invent*, coinvent*<br>co-develop*, codevelop*<br>co-produc*, coproduc*<br>“user-centred design”, “user-centered design”<br>“consumer engagement”<br>“consumer participation”<br>“consumer involvement”<br>“consumer co-leadership”<br>“consumer consultation”<br>“participatory design”<br>“collaborative design”<br>“human-centred design”, “human-centered design”<br>“experience-based design”<br>“experience-based co-design’", “experience-based codesign”<br>“community-based participatory research”<br>“community involvement”<br>“patient consultation”<br>“patient engagement”<br>“patient input”<br>“stakeholder participation”<br>“stakeholder engagement”<br>“product design”<br>“participatory framework”<br>“participatory action research”<br>“participatory research”	cancer*<br>neoplasm*<br>tumo#r*<br>oncolog*<br>carcinoma*<br>haematolog*<br>hematolog*<br>malignan*<br>“cancer care”<br>"oncologic care"<br>"cancer rehabilitation"<br>"cancer survivorship"<br>"cancer diagnos*"

Supplementary Table 2: Overview of the included studies (N= 51)

Study/year	Country	Co-designed initiative	Study dates	Duration of co-design initiative	Setting	Initiative type	Purpose of co-design initiative	Funding source	Target group	Source of consumer participants	Consumer stakeholder engagement type[1]
Al-Itejawi 2016[19]	Netherlands	Shared decision making patient decision aid (unnamed initiative)	NR	NR	Outpatient	Patient decision aid	Facilitate patient participation and support prostate cancer patients in making specific and deliberative choices for their anti-cancer treatment by providing information on the options and outcomes relevant to them.	No funding received	Men with prostate cancer	National prostate cancer charitable organisations	Co-production
Ankolekar 2019[20]	Netherlands	Online shared decision making patient decision aid (unnamed initiative)	NR	NR	Outpatient	Patient decision aid	To provide accurate and balanced information about the main treatment options for prostate cancer and a means for patients to discover and communicate their preferences with HCPs during the consultation.	The European Program and Health Foundation	Men with prostate cancer	Former patients (source not specified)	Co-production

Azizoddin 2021[26]	USA	Smartphone Technology to Alleviate Malignant Pain (STAMP)	NR	NR	Outpatient	Application	To develop comprehensive cancer pain education materials formatted for mHealth, and to leverage the unique potential of technological solutions to deliver education to patients in a way that is responsive to their personalized and unique pain management needs in the moment they need it.	National Institutes of Health	Patients experiencing cancer pain	Patient Advisory Council from an academic cancer centre	Co-production
Børøsund 2018[28]	Norway	StressProffen: Multimodal app-based intervention	2015-2016	16 months	Outpatient	Application enhanced with one in-person session and one follow-up telephone call	Stress management self-help program	Norwegian Cancer Society	Patients with cancer	Oslo University hospital and collaborating networks, Norwegian Cancer Society and social media	Co-production
Bricker 2020[29]	USA	Quit2Heal: app-based intervention	NR	NR	Outpatient	Application	Help cancer patients quit smoking	Consumer Value Store Health Foundation, National Cancer Institute, and	Patients with cancer who smoke	Two large academic cancer	Co-production

								National Institute on Drug Abuse			
Hochstenbach 2023[38]	Netherlands	PROSPECT (prostate cancer decision aid for side effects): Shared decision making patient decision aid	2020	9 months	Outpatient	Patient decision aid	Personalise patient decision aid to facilitate more effective shared decision making and communication for men with prostate cancer, with a primary focus of deciding from treatment options.	Zorginstituut Nederland	Men with prostate cancer	Prostate cancer patient organisation	Co-production
Holch 2016[41]; Holch 2017[40]; Warrington 2019[80]	UK	eRAPID programme (electronic patient self-Reporting of Adverse-events: Patient Information and aDvice): Patient reported outcomes platform	NR	9+ months	Outpatient	Software (computers or tablets)	A system for patients to self-report and manage adverse events during and after cancer treatment.	National Institute for Health Research	Cancer survivors (during and post-treatment) and their healthcare team	Institute of Oncology at St James's University Hospital	Co-production
Izard 2014[43]; Hartzler 2016[36]	USA	Quality of Life (QOL) Tracker: Patient reported outcome dashboard (named "Men	NR	NR	Outpatient	Digital resource	Illustration via digital dashboard of personalised trends in prostate cancer patients health-related quality of life	National Cancer Institute, National Institutes of Health and Fred	Men with prostate cancer and their treating HCPs	Local prostate cancer support groups and University of Washington urology clinics	Co-production

		like Me" in other sources)					(as measured by the Expanded Prostate Cancer Index) to ultimately enhance shared-decision making during outpatient consultations with HCPs	Hutchinson Cancer Research Center			
Miller 2020[51]	UK	Prototype of a digital remote monitoring application (unnamed initiative)	NR	NR	Mixed (inpatient and outpatient)	Application prototype	Support patients for the first 30 postoperative days following colorectal cancer surgery	MRC "Confidence in Concept" award	Patients who had colorectal cancer surgery	Clinical contacts	Co-production
Monteiro-Guerra 2020[51]; Monteiro-Guerra 2020[52]; Signorelli 2022[68]	Spain and Ireland	Physical activity intervention (unnamed)	2018-2020	20 months	Community	Application	Personalised physical activity coaching that targets the needs of breast cancer survivors at the group and individual level	European Union's Horizon 2020 research and innovation program	Breast cancer survivors (post primary curative treatment)	Oncology clinic	Co-production
Noordman 2017[55]; Noordman 2017b[56]	Netherlands	ListeningTime website	2015	5 months	Outpatient	Website, communication resource	To help older patients with cancer and their oncological HCPs better prepare for encounters with each other via education and interactive components.	Dutch Cancer Society	Older adults with cancer, oncology HCPs	Patient organisation NFK (Nederlandse Federatie van Kankerpatiënten organisaties)	Co-production

										and National Panel of people with Chronic illness or Disability (NPCD)	
O'Malley 2020[59]	USA	Extended Cancer Education for Longer-term Survivors eHealth self-management tool (e-EXCELS)	2014-2015+	5+ months	Outpatient	Website, lifestyle intervention	Improve adherence to follow-up care guidelines for cancer survivors (post-treatment)	National Cancer Institute	Long term cancer survivors	Community-based survivorship organisations, local oncology, and primary care practices and the Rutgers University Faculty and Staff online bulletin	Co-production
O'Malley 2022[58]	USA	Extended Cancer Education for Longer-term Survivors health-coaching (EXCELS-hc) intervention	2014-2015+	5+ months	Outpatient	Lifestyle intervention	Promote accurate processing of recurrence risk and other post-treatment health-related risks, identify emergent or ongoing treatment symptoms, align beliefs and expectations about need for risk-based preventative care and management of existing sequelae	None reported	Cancer survivors (post-treatment or maintenance therapy)	Community-based survivorship organisations, local oncology, and primary care practices and the Rutgers University Faculty and Staff online bulletin	Co-production

							based on current evidence, and identify action steps to encourage guideline concordant follow-up care				
Prince 2019[61]	Canada	Bridges: Web-based tool for chemotherapy support	2014 - 2015	11 months	Outpatient	Digital portal for healthcare delivery and education	Facilitate remote management of chemotherapy-related toxicities and symptoms online via a patient and health care provider interactive and informative portal	University of Toronto, Princess Margaret Cancer Centre Foundation, the Ontario Institute for Cancer Research	Patients receiving outpatient chemotherapy, their carers, and HCPs	Medical oncologist or Cancer Care Ontario Patient and Family Advisory Council	Co-production
Renehan 2022[63]	Australia	Information Guide about Scalp Cooling: Digital information booklet	2019 - NR	2+ months	Outpatient	Education resource, digital	Better prepare and inform women considering scalp cooling for the prevention of hair loss during chemotherapy	No funding received	Newly diagnosed cancer patients at risk of chemotherapy induced alopecia / hair loss	Hospital (name not reported)	Co-production
Roth 2022[64]	USA	Health State Description (HSD) for low-risk thyroid cancer (unnamed)	2021 - 2022	11 months	Outpatient	Educational resource	Describe the disease state (low risk thyroid cancer) in plain language to enable consumers to state their treatment preferences	American College of Surgeons Fellowship	Low risk thyroid cancer survivors (pre, active, and post-treatment)	Professional networks and Thyroid Cancer Survivors' Association	Co-production

Saxton 2022[65]; Saxton 2022[44]	UK	Lifestyle behaviour change intervention for weight-loss (unnamed)	2018 - 2019	5 months	Community	Lifestyle intervention	Support women recovering from oestrogen receptor-positive breast cancer treatment to lose weight via a long-term peer-support enhanced educational and behaviour change lifestyle intervention	Yorkshire Cancer Research	Women recovering from primary oestrogen receptor-positive breast cancer treatment	Patients attending routine clinical visits (health services not specified)	Co-production
Sparidaens 2022[70]	Netherlands	Online information material (unnamed initiative)	2016 - 2017	24+ months	Outpatient	Website, educational resource	Provide information concerning fertility and early menopause for breast cancer patients.	Radboud Oncology Fund	Young female breast cancer survivors	University hospitals	Co-production
Sungur 2020[72]; Yilmaz 2022[84]	Netherlands	'Conversation Starter', an oncology module for 'Health Communicator' or independent use	2017 - NR	7+ months	Outpatient	eHealth resource	Improve participation and satisfaction with care by migrant cancer patients via improved communication with HCPs and culturally-appropriate educational resources	KWF Kankerbestrijding	Turkish-Dutch and Moroccan-Dutch migrant patients with cancer	Through prominent figures within Turkish and Moroccan Communities, hospitals, general practitioners, health centers and patient organisations	Co-production
Tang 2020[73]	Australia	Prehabilitation model of care (named tentatively	2018	3 months	Outpatients	Design for a program	Provide prehabilitation to prostate cancer patients	Western & Central Melbourne	Men with prostate cancer before primary treatment	Two health services	Co-production

		"Locker Room" or "Co-PreP")						Integrated Cancer Service			
Timmerman 2016[76]; Timmerman 2017[75]	Netherlands	Telehealthcare application for lung rehabilitation (unnamed)	NR - 2016	24+ months	Outpatients	Telehealth app	Improve post-surgery rehabilitation and physical activity in resected NSCLC survivors	Dutch Cancer Society	NSCLC surgery patients	Through health care professionals known to the authors, advertising on the website of the Dutch lung cancer association and Netherlands Cancer Institute	Co-production
Tsangaris 2022[77]	USA	imPROVE: Patient reported outcome platform	2018 - 2021	35 months	Outpatient	Application (patients), digital dashboard (HCPs)	Support a collaborative care approach through adaptive tailored feedback, optimising care for patients, and empowering and enabling patient to their own care; and to inform clinical decision making, quality improvement, and research.	Department of Surgery at Brigham and Women's Hospital	Breast cancer survivors (during and post-treatment) and their HCPs	Through medical professionals at scheduled clinic visits and breast cancer advisory groups	Co-production
Tseng 2021[78]	Taiwan	Online shared decision making aid (unnamed initiative)	2015 – 2018+	24+ months	Outpatient	Patient decision aid	Provide medical information and help the patient explore and compare	Ministry of Science and Technology, Taipei Veterans	Women with breast cancer	Not described	Co-production

							treatment options, assess the patient's values and preferences, and reach a collective decision on fertility preservation for breast cancer patients	General Hospital, Melissa Lee Cancer Foundation			
vanStrien-Knippenberg 2022[79]	Netherlands	Shared decision making patient decision aid (unnamed initiative)	NR	NR	Outpatient	Patient decision aid	A total package of decision-relevant information about adjuvant breast cancer treatment	Health Holland and Dutch Insurer CZ	Women with breast cancer	Responders to a previous survey study (not references and recruitment details not described)	Co-production
Wolpin 2015[81]	USA	Electronic Self-Report Assessment for Cancer (ESRAC) 2.0: Computerised symptom and quality of life information system	2008	8 months	Outpatient	Online computer portal	Encourage patients to self-monitor symptom and quality of life information, access educational information about self-care, and receive coaching on how to communicate symptoms and quality of life information with HCPs.	National Institutes of Health	Any patient with cancer and their HCPs	Flyers in the outpatient oncology centre	Co-production
Aronoff-Spencer 2022[21]	USA	MyPath app and service model	2017-2019	23 months	Online	Application and model of care	A system to facilitate communication about cancer-related distress among	Industry funding	Patients with cancer and their family and treating HCPs	Recruited onsite at healthcare facilities (specific	Co-design

							patients, caregivers, and providers.			facilities not named)	
Austin 2020[23]	USA	Patient-centred pathology report template (unnamed initiative)	NR	NR	Outpatient	Report template	Improve patient-centredness of pathology reports which deliver key diagnostic information about a cancer diagnosis	American Cancer Society	Patients recently diagnosed with cancer	Local cancer support groups and study investigator's clinical practices	Co-design
Austin 2021[24]; Austin 2022[25]	Netherlands	Compas-Y: mobile self-compassion intervention	2018-2020	24 months	Community	Application	Improve self-compassion by patients newly diagnosed with cancer	Dutch Cancer Society	Patients recently diagnosed with cancer	Two participating hospitals	Co-design
Grant 2021[33]; Leske 2022[47]	Australia	Healthy Living after Cancer Online	2019-2020	18 months	Online	Online intervention	NR	Cancer Council South Australia	Cancer survivors (completed curative treatment)	Cancer Council South Australia	Co-design
Hall 2022[35]	UK	ACTION: Acceptance and commitment therapy program for medication adherence in breast cancer survivors	NR	NR	Outpatient	Individual group therapy	Acceptance and commitment therapy-based intervention to support adjuvant endocrine therapy medication decisions and quality of life in breast cancer survivors	Yorkshire Cancer Research	Breast cancer survivors (post-primary treatment) receiving adjuvant endocrine therapy	Cancer support groups and social media	Co-design
Hochstenbach 2017[37]	Netherlands	eHealth-enhanced intervention	NR	9 months	Outpatient	Multimodal eHealth (iPad app for	Facilitate nurses to support self-management of	Dutch Cancer Society	Patients experiencing cancer pain	Medical contacts	Co-design

		(unnamed initiative)				patients; website portal for nurses; some in-person components)	outpatients experiencing cancer pain				
Hoffman 2019[39]	USA	"Considering Breast Reconstruction after Mastectomy: A Video and Workbook for Women with Breast Cancer": Patient decision aid	NR	9 months	Outpatient	Patient decision aid	Assist patient decision making after unilateral mastectomy in breast cancer survivors by educating survivors and preparing them for informed conversations (shared decision making) with their treating team.	The University of Texas MD Anderson Cancer Center Duncan Family Institute for Cancer Prevention and Risk Assessment	Women who have had or planning to have a mastectomy as breast cancer treatment	Not reported	Co-design
Kemp 2018[45]; Beatty 2021[27]	Australia	Finding My Way-Advanced (FMW-A): Online support program	NR	NR	Community	Online intervention	Support women living with metastatic breast cancer	Cancer Council South Australia and Flinders Foundation	Women with advanced breast cancer	Tertiary public hospital and Flinders Centre for Innovation in Cancer	Co-design
Lipson-Smith 2019[48]	Australia	SecondEars: Consultation audio-recording mobile application	2016 - 2017	5+ months	Outpatient	Application	To provide patients with the permission and means to audio-record their consultations	Peter MacCallum Cancer Centre Foundation and the Victorian Managed Insurance Authority	Patients, family, (HCPs, hospital administrators)	Not reported	Co-design
Milton 2022[52]	UK	All Together Active: Social	2018 - NR	NR	Community	Lifestyle intervention	Social intervention to support people affected by a cancer	Cancer Research UK Population	Cancer survivors	Local cancer charity	Co-design

		physical activity intervention					diagnosis to be physically active	Research Committee and BUPA Foundation	(active and post-treatment)		
O'Gara 2022[57]	UK	SafeSpace: Virtual reality compassionate mind training intervention	NR	6 months	Outpatients	Digital psychological intervention	Support people undergoing cancer treatment in the clinical setting via rapid access to safe, calm, and soothing environments	Macmillan Cancer Support	Patients receiving anti-cancer treatment	Specialist centre	Co-design
Petit-Steeghs 2021[60]	Netherlands	Health education intervention via simplified care pathway for urological cancer patients (unnamed)	2015 - 2017	15 months	Outpatients	Digital health intervention	Provide and support interpretation of tailored educational information to patients with urological cancer, facilitate shared decision-making, and facilitate self-management.	Netherlands Organisation for Health Research and Development	Patients with urological cancer	Patient organisations	Co-design
Schmidt 2020[66]	Switzerland	Switzerland adapted Cancer Thriving and Surviving(CTS) program	2016 - 2017	7 months	Outpatient	Self-management intervention	Peer-led program to impower breast cancer survivors in self-management skills	Swiss Cancer Research	Breast cancer survivors post primary treatment	Swiss breast centres	Co-design
Thomas 2019[74]	USA	Strong Together, self-advocacy serious game	NR – 2017 - NR	2+ months	Outpatient	Serious game	Improve self-advocacy skills for women with advanced cancer	Beckwith Institute Clinical Transformation	Women with advanced cancer	Not described	Co-design

								Program and Rockefeller University			
Yan 2023[83]	China	Character strengths-based intervention for Chinese women with breast cancer (unnamed initiative)	NR	27 months	Outpatient	Clinician-delivered psychological intervention with written booklet	To enhance self-esteem and quality of life and alleviating depression amongst Chinese patients with breast cancer.	None reported	Women with breast cancer	Not described	Co-design
Ashmore 2020[22]	UK	Digital health intervention (unnamed initiative)	NR	NR	Community	Application prototype	Support women who have had treatment (radiotherapy) for gynaecological cancer	Medical Research Council	Women with gynaecological cancer treated via radiotherapy and their family and treating HCPs	Local Cancer Alliance Partnership Group and follow up clinics (details not described)	Co-creation
Eggly 2013[30]	USA	Question prompt list (unnamed initiative)	2011	9+ months	Outpatient	Resource	To be used in a minority population (focus on Black Americans) of cancer patients facing a discussion with their oncologist about chemotherapy treatment.	National Cancer Institute	Black patients recently diagnosed with cancer considering chemotherapy as a treatment option	Residents of the surrounding community (further source details not described)	Co-creation
Foster 2015[32]	UK	RESTORE: Online intervention	2009-2012+	36 months	Community	Online intervention	Enhance people's confidence to live with cancer-related	Macmillan Cancer Support	Cancer survivors (completed	Not reported (development phase), local	Co-creation

							fatigue following primary cancer treatment		curative treatment) experiencing fatigue	newspapers, advertising on project website and the user reference group from the development phase (user testing phase)	
Gunn 2013[34]; Fennell 2017[31]	Australia	Country Cancer Support: Informational website	2010-2015	60 months	Community	Website, educational resource	Rural-specific online information on 'how to cope and who can help' when faced with a diagnosis of cancer; reduce users feelings of distress, perceived isolation, and encourage psychosocial help seeking.	Cancer Council Australia, Cancer Council South Australia, Country Health SA	Patients with cancer who live rurally	Cancer Council SA, local media and personal contacts	Co-creation
Hyatt 2021[42]	Australia	Evidence-based videos	2018	8 months	Online	Online videos, educational resource	Address immunotherapy-related uncertainty and assist with effective identification and reporting of side effects for patients and their family carers	Cancer Nurses Society Australia	Patients about to receive immunotherapy anti-cancer treatment and their family carers	Peter MacCallum Cancer Centre	Co-creation

Loeliger 2021[49]; Loeliger 2023[50]	Australia	CanEAT Pathway: Cancer nutrition care pathway	2018-2019	14 months	Outpatient	Patient resource	Guide and improve the provision of consistent and evidence-based nutrition care of patients throughout the cancer care continuum	Victorian Government	Cancer survivors (pre, during, and post-care)	Cancer and consumer organisations, health service consumer networks and health services	Co-creation
Rackerseder 2022[62]; Krieger 2022[46]	Germany	Integrated Cross-Sectoral Psycho-Oncological Patient Information Materials	2019-2021	10 months	Outpatient	Educational resources (five resources; online and hardcopy)	Educational information (website...) about a federally funded oncology support program (12-month psychosocial and psychotherapeutic isPO (Integrated Cross-Sectoral Psycho-Oncological) program offered to newly diagnosed adult cancer patients, parallel to their biomedical therapeutic treatment')	No funding received	Newly diagnosed cancer patients	Cancer self-help groups	Co-creation
Shemesh 2022[67]	Australia	BroSupPORT web portal: Patient reported outcome dashboard	NR	NR	Outpatient	Digital resource	Provide information, feedback, and resources customised to the needs of Australian men with	Victorian Agency for Health Information	Prostate cancer survivors (during and post-treatment)	Cancer Council Victoria, Prostate Cancer Foundation Australia,	Co-creation

							prostate cancer (post diagnosis or treatment)			Prostate Cancer Outcomes Registry	
Singleton 2021[69]	Australia	Bank of evidence-based text-message content (unnamed)	2018-2019	9 months	Community	mHealth resource	A bank of text-message content aimed to support women's mental and physical health after breast cancer treatment.	No funding received	Breast cancer survivors (post-primary treatment)	Breast Cancer Network Australia, Westmead Breast Cancer Institute	Co-creation
Sun 2021[71]	UK	Digital health intervention (unnamed initiative)	NR	NR	Outpatient	Application	Improve cancer patient experiences and their quality of life, reduce nursing administrative workload, and improve health service efficiency as well as health outcomes	Macmillan Cancer Support	Patients with oesophageal cancer, their carers, and HCPs	Local hospital and university networks, referrals from health professionals	Co-creation
Woodard 2018[82]	USA	Pathways: A fertility preservation patient decision aid website for young women with cancer	NR	NR	Outpatient	Patient decision aid	Prepare women for informed discussions with their oncology providers about their potential fertility risk, options for fertility preservation, and referral to a fertility specialist, if desired. To provide a balanced and understandable	The University of Texas MD Anderson Cancer Center Duncan Family Institute for Cancer Prevention and Risk Assessment	Female cancer patients and survivors (pre or post treatment)	Not described	Co-creation

							overview of the fertility preservation and alternative family-building options to support informed discussions about whether to pursue fertility preservation treatments.				

[1]Definition from Vargas et al.
